# Identification of miRNA Signature in Breast Cancer to Predict Neoadjuvant Chemotherapy Response

**DOI:** 10.3389/pore.2021.1609753

**Published:** 2021-04-30

**Authors:** Ai-Yan Xing, Bin Wang, Yu-Hong Li, Xu Chen, Ya-Wen Wang, Hai-Ting Liu, Peng Gao

**Affiliations:** ^1^ Department of Pathology, School of Basic Medicine Science, Shandong University, Jinan, China; ^2^ Department of Pathology, Shandong University Qilu Hospital, Jinan, China; ^3^ Department of Surgery, The First Affiliated Hospital of Shandong First Medical University, Jinan, China; ^4^ Department of Pathology, Liaocheng People’s Hospital, Liaocheng, China; ^5^ Department of Breast Surgery, Shandong University Qilu Hospital, Jinan, China

**Keywords:** breast cancer, microarray, miRNA signature, neoadjuvant chemotherapy, drug resistance

## Abstract

**Background:** Chemotherapy failure causes high breast cancer recurrence and poor patient prognosis. Thus, we studied a cohort of novel biomarkers to predict chemotherapeutic response in breast cancer. In this study, miRNA expression profiling was performed on 10 breast cancer punctured specimens sensitive to chemotherapy (MP grade 4, 5) and 10 chemotherapy resistant (MP grade 1). Differentially expressed miRNAs were verified by qRT-PCR in 60 initial samples, 59 validated samples and 71 independent samples. A miRNA signature was generated using a Logistic regression model. A receiver operating characteristic (ROC) test was used to assess specificity and sensitivity of single miRNA and miRNA signature. Target genes regulated by miRNAs and their involved signaling pathways were analyzed using GO enrichment and KEGG software. MiRNAs expression were separately compared with ER, PR, HER2 immunohistochemical staining and different drugs. qRT-PCR showed that the high expression of miR-23a-3p, miR-200c-3p, miR-214-3p and the low expression of miR-451a and miR-638 were closely related to chemoresistance. According to the formula for calculating the drug resistance risk, patients in the high-risk group were more likely to develop chemotherapy resistance than the low-risk group. Bioinformatics analysis showed that 5 miRNAs and target genes are mainly involved in p53, ubiquitin-mediated proteolysis, mTOR, Wnt, cells skeletal protein regulation, cell adhesion and ErbB signaling pathways. miR-451a expression was associated with ER, HER-2 status and anthracyclines. A miRNA signature of chemotherapeutic response may be clinically valuable for improving current chemotherapy regimens of individual treatment for patients with breast cancer.

## Introduction

According to the American *Cancer* Society, 244,660 breast cancer cases of women were estimated in 2016, and of these almost 40,450 people died from these cancers [1]. Thus, breast cancer kills more women than any other cancer world-wide. Now, surgery and chemotherapy are chief first-line therapies for breast cancer [2], but high recurrence and metastasis reduce the patients’ survival attributing to poor chemotherapeutic effect. Thus, we must identify novel biomarkers to predict chemotherapeutic response. Neoadjuvant chemotherapy (NAC), which can reduce primary tumor size prior to surgery, is widely used for breast cancer and chemotherapeutic response can be assessed using Miller and Payne (MP) grades after NAC [3]. Thus, it was feasible and innovative to analyze breast cancer samples after NAC to evaluate chemotherapeutic response and predict the best therapy.

Research suggests that microRNAs (miRNAs) are involved in cancer initiation and progression. miRNAs, small non-coding RNAs that negatively regulate genes by combining to the 3’ untranslated region (3’UTR) of target messenger RNAs (mRNAs), are reported to be critical for drug resistance. miR-221/222, upregulated in HER2/neu-positive primary human breast cancer tissues, mediates tamoxifen resistance by targeting cell cycle inhibitor p27Kip1 [4]. miR-328 negatively regulated expression of breast cancer resistance protein (BCRP/ABCG2) to increase drug sensitivity to mitoxantrone [5]. Pogribny’s group identified 46 upregulated and 57 downregulated expressed miRNAs between MCF-7/CDDP and parental MCF-7 cells using microarray cluster analysis [6], suggesting a potential role for these miRNAs in cisplatin-based resistance. However, one biomarker or data from one cell line (representing one case) is not sufficient for predicting a chemotherapeutic response. Thus, integrating multiple biomarkers from larger clinical samples will have better predictive value.

In this study, we identified a signature of five differentially altered miRNAs between chemosensitive and chemoresistant breast cancer tissues, based on miRNA expression profiling. We sought to develop a miRNA signature to predict chemotherapeutic response using a training set and the predictive accuracy of signature was assessed with a testing patient group and validated with an independent patient group.

## Materials and Methods

### Clinical Specimens and Study Design

**FIGURE 1:**
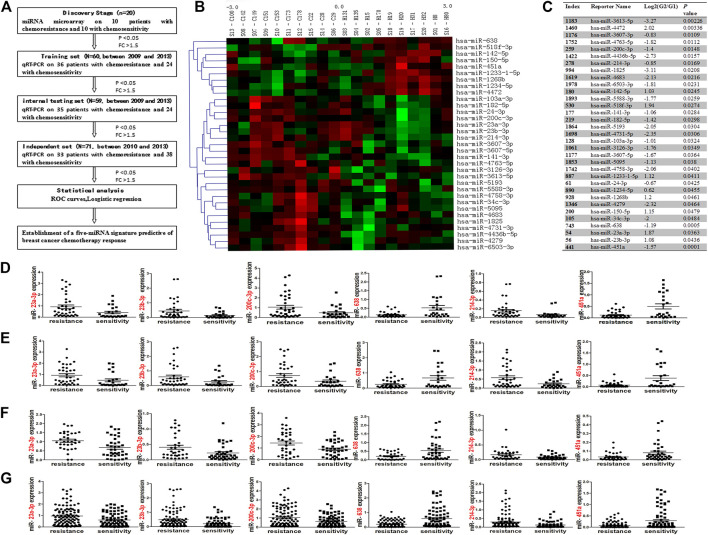
A workflow of the experimental design was showed **(A)**. Cluster analysis in chemoresistant **(C)** and chemosensitive **(H)** breast cancer tissues **(B)**. Hierarchical clustering of 33 miRNAs differentially expressed in 20 breast cancer patients, including miR-638, miR-23a-3p, miR-23b-3p, miR-200c-3p, miR-214-3p and miR-451a. Red: up-regulated; green: down-regulated miRNAs in the chemoresistant group. Samples are in columns; miRNAs are in rows. Expression values ranged from −3 to +3 log2. **(C)** 33 miRNAs are significantly expressed between chemoresistant (G1) and chemosensitive (G2) groups. Mean fold-change of chemosensitive vs. chemoresistant groups at log2 (G2/G1). qRT-PCR results showed that miR-23a-3p, miR-23b-3p, miR-200c-3p, and miR-214-3p were significantly up-regulated, and miR-638 and miR-451a were significantly downregulated in the chemoresistant group in the training set [**(D)**; *p* = 0.0259; 0.0379; 0.0354; 0.0367; 0.001; 0.002], internal testing set [**(E)**; *p* = 0.0292; 0.0486; 0.0283; 0.118; 0.0036; 0.013], in the independent set [**(F)**; *p* = 0.0026; 0.0195; 0.0077; 0.0162; 0.0036; 0.0209] and combined set (including training set, internal testing set and the independent set; **(G)**: *p* = 0.0015; 0.0003; 0.0037; 0.003; 0.001; 0.001). The data was represented as the mean and SE (standard error).

Tissues (*N* = 190) formalin-fixed paraffin-embedded (FFPE) needle biopsy samples of breast cancers before chemotherapy assessed for miRNA expression. Specimens (*N* = 119) were obtained from Qilu Hospital, Shandong University, Jinan, China, between 01/2009 and 12/2013. 20 cases of these 119 samples, including 10 chemosensitive (MP grade 4 and 5) and 10 chemoresistant (MP grade 1) breast cancer needle biopsy tissues, were selected to perform a microRNA microarray. Then we randomly divided 119 specimens (including 99 naive cases and 20 cases for the microarray analysis) into a training set of 60 samples and a validation set of 59 samples with a computer-generated allocation sequence. We sought to identify a predictive miRNA signature of chemotherapeutic response from the training set and tested it with the internal testing set. To validate the predictive value of the miRNA signature in different populations, other 71 samples were collected as an independent set from Liaocheng People’s Hospital, Liaocheng, China, between 01/2010 and 12/2013. Clinicopathological characteristics of all samples were available from pathology reports and patient files (See Table 1). The Ethics Committee of Shandong University (approval code: 201101015) approved this study and the procedures involving human subjects were in accordance with the Declaration of Helsinki. A workflow of the experimental design was showed in Figure 1A.

**TABLE 1:** Clinical characteristics of patients in training, internal testing, and independent set.

	Training set (*N* = 60)	Internal testing set (*N* = 59)	Independent set (*N* = 71)
Age			
≤40	9 (15%)	16 (27.1%)	16 (22.5%)
41–60	39 (65%)	36 (61.0%)	43 (60.6%)
≥61	12 (20%)	7 (12.9%)	12 (16.9%)
Lymph node metastasis			
absent	14 (23.3%)	14 (23.7%)	24 (33.8%)
present	46 (76.7%)	45 (76.3%)	47 (66.2%)
T Stage			
T1	11 (18.3%)	17 (28.8%)	10 (14.1%)
T2	42 (70%)	31 (52.5%)	45 (63.4%)
T3	7 (11.7%)	11 (18.7%)	16 (22.5%)
*N* stage			
N0	14 (23.3%)	14 (23.7%)	24 (33.8%)
N1	16 (26.7%)	8 (13.6%)	17 (23.9%)
N2	17 (28.3%)	21 (35.6%)	16 (22.5%)
N3	13 (21.7%)	16 (27.1%)	14 (19.7%)
TNM stage			
I	3 (5%)	3 (5.1%)	6 (8.5%)
II	21 (35%)	17 (28.8%)	29 (40.8%)
III	32 (53.3%)	38 (64.4%)	36 (50.7%)
IV	4 (6.7%)	1 (1.7%)	0 (0)
Chemotherapy drug			
AC	11 (18.3%)	4 (6.8%)	2 (2.8%)
CTF	18 (30%)	13 (22.0%)	2 (2.8%)
AT	29 (48.3%)	39 (66.1%)	63 (88.7%)
CMF	2 (3.3%)	3 (5.1%)	4 (5.6%)
Chemotherapy cycles			
≤4	45 (75%)	38 (64.4%)	43 (60.6%)
>4	15 (25%)	21 (33.6%)	28 (39.4%)
Chemotherapy assessment			
resistance	36 (60%)	35 (59.3%)	33 (46.5%)
sense	24 (40%)	24 (40.7%)	38 (53.5%)
Histological grade			
I	4 (6.7%)	1 (1.7%)	7 (9.9%)
II	28 (46.7%)	44 (74.6%)	33 (46.5%)
III	28 (46.7%)	14 (23.7%)	31 (43.7%)
ER			
negative	17 (28.3%)	18 (30.5%)	32 (45.1%)
positive	43 (71.7%)	41 (69.5%)	39 (54.9%)
PR			
negative	25 (41.7%)	24 (40.7%)	47 (66.2%)
positive	35 (58.3%)	35 (59.3%)	24 (33.8%)
HER2			
negative	31 (51.7%)	17 (28.8%)	25 (35.2%)
positive	29 (48.3%)	42 (71.2%)	46 (64.8%)
P53			
negative	24 (40%)	20 (33.9%)	19 (26.8%)
positive	36 (60%)	39 (66.1%)	52 (73.2%)
Ki-67			
≤14%	16 (26.7%)	13 (22.0%)	22 (30.9%)
>14%	44 (73.3%)	46 (78.0%)	49 (69.1%)
Molecular subtype			
HER2-overexpress	10 (16.7%)	15 (25.4%)	24 (33.8%)
Basal-like	7 (11.7%)	3 (5.1%)	7 (9.9%)
Luminal A	24 (40%)	14 (23.7%)	10 (14.1%)
Luminal B	19 (31.7%)	27 (45.8%)	30 (42.3%)

AC: Anthracycline and Cyclophosphamide; CTF: Cyclophosphamide, Taxol and Fluorouracil; AT: Anthracycline and Taxol; CMF: Cyclophosphamide, Methotrexate and Fluorouracil.

### Miller and Payne Response Assessment

All patients with breast cancer in this study received NACs of different cycles (2–8 cycles) after diagnosis by biopsy and then tumor resection. Pathological MP assessment was evaluated based on reduced tumor cellularity of resection samples and comparison with pre-treatment needle biopsy tissues. After neoadjuvant chemotherapy, we usually cut a section every 0.5–1 cm of tumor bed according to the tumor size and make into HE slides for MP response assessment. MP response assessment was determined by two pathologists (P Gao and Y-H Li). Patients were divided into a chemoresistant group (grades 1–2) and a chemosensitive group (grades 3; 4 and 5) according to MP grades [7]. The difference was compared between chemosensitive and chemoresistant groups in our study. Chemosensitivity occurred in 24 (40.00%) cases in the training set, 24 (40.67%) cases in the internal testing set, and 38 (53.52%) cases in the independent set.

### μParaflo™ MicroRNA Microarray Assay

We measured miRNA expression in breast cancer biopsy samples using microarray. The miRNA microarray chip covered all miRNAs in the miRBase database version 19.0 (http://www.mirbase.org/; including 2019 miRNAs), and was a product of LC Sciences (Houston, TX, United States). After miRNAs were 3’-extended with a poly(A) tail and labeled with an oligonucleotide tag, a hybridization assay was performed overnight on a μParaflo™ microfluidic chip (Atactic Technologies, Houston, TX, United States). Then, tag-conjugating Cy3 dye was stained and fluorescent signals were collected and digitized using Array-Pro image analysis software (Media Cybernetics, Bethesda, MD, United States). Data were analyzed by first subtracting the background and then normalizing the signals using a LOWESS filter (Locally weighted Regression). Comparison between chemoresistant and chemosensitive groups, the differentially expressed miRNAs were selected according to fold change (FC > 1.5) and *p* value (*p* < 0.05).

### Data Processing

After background subtraction and normalization, significantly differential-expressed miRNAs were identified. Distinct miRNA expression between two groups was confirmed *via* hierarchical clustering analysis. In addition, target prediction algorithms Targetscan (www.targetscan.org), miRanda (http://www.microrna.org/) and PITA (http://genie.weizmann.ac.il/pubs/mir07/mir07_data.html) were used to identify targets of dysregulated miRNAs. Gene Ontology (GO) and Kyoto Encyclopedia of Genes and Genomes (KEGG) pathway enrichment analysis of the specific miRNAs targets were performed using the web-based tool StarBase (http://starbase.sysu.edu.cn/). The potential functional network of the selected miRNAs and their targets were constructed by Cytoscape software (http://www.cytoscape.org/). The microarray data was deposited in ArrayExpress (GSE73736) according to minimum information about a microarray experiment (MIAME) guidelines.

### miRNA Extraction and Real-Time Quantitative PCR (qRT-PCR)

**FIGURE 2:**
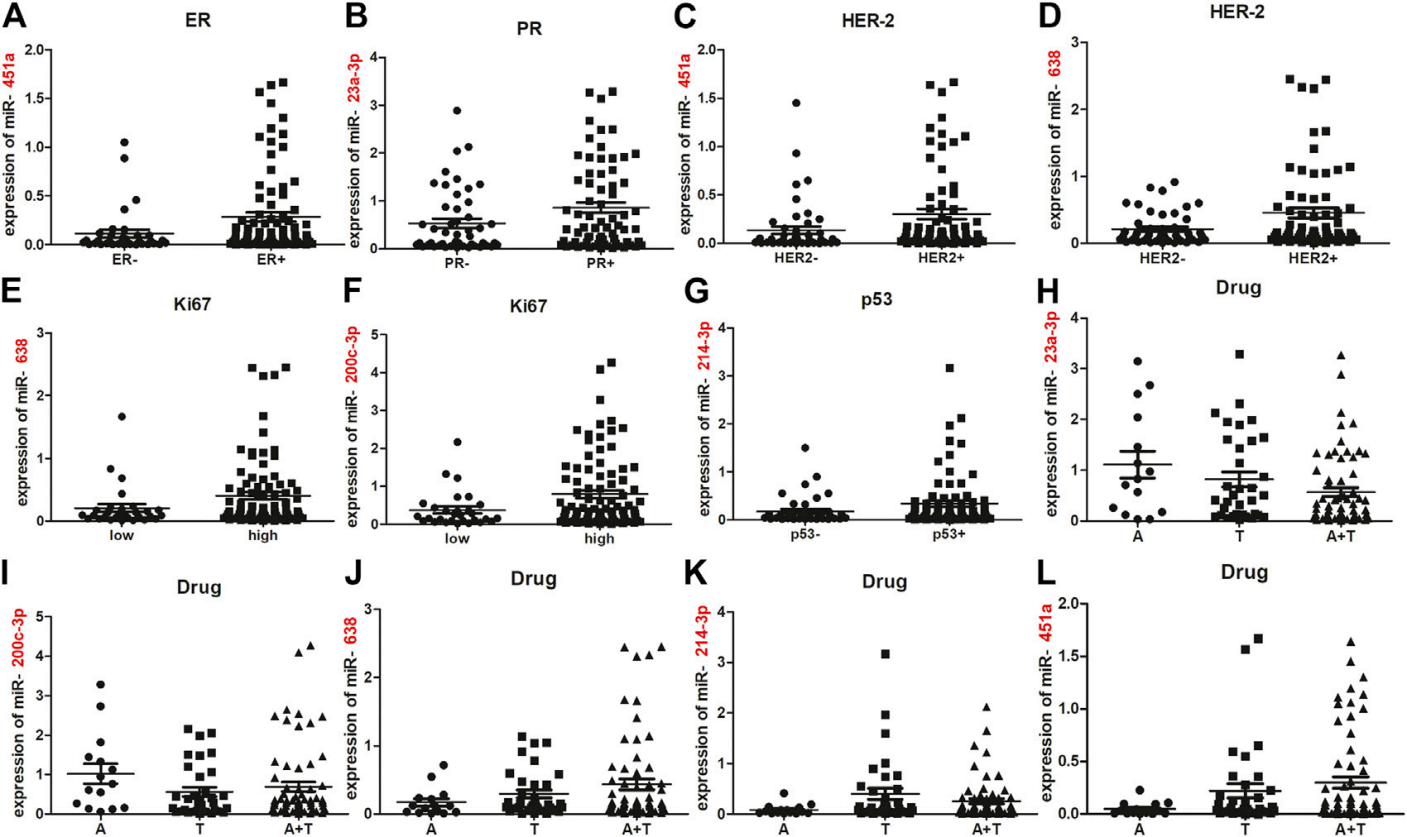
Association between five deregulated miRNAs and endocrine features or chemotherapy drugs. miR-451a was less expressed in ER-negative cases compared to ER-positive cases [**(A)**; *p* = 0.0296]. miR-23a expression was greater in PR-positive cases compared to PR-negative cases [**(B)**; *p* = 0.0367]. miR-638 and miR-451a were down-regulated in HER2-negative cases, compared with HER2-positive cases [**(C, D)**; *p* = 0.0219; 0.0117]. Compared with Ki67-low cases, more expression of miR-638 and miR-200c-3p were observed in Ki67-high cases [**(E, F)**; *p* = 0.017; 0.0233]. miR-214-3p expression was increased in p53-positive vs. p53-negative cases [**(G)**; *p* = 0.0213]. miR-23a-3p expression in A and T groups were higher than in the AT group [**(H)**; *p* = 0.0393; 0.0430]. Upregulated miR-200c-3p was observed in A group, compared with T and AT groups [**(I)**; *p* = 0.0177, 0.0361]. miR-214-3p was upregulated in T group, compared with A and AT groups [**(K)**; *p* = 0.0254, 0.0373]. Reduced expression of miR-638 occurred in the A group, compared with T and AT groups [**(J)**; *p* = 0.019, 0.0162]. Significantly step-upregulated expression of miR-451a occurred in A, T and AT groups **(L)**. Significant difference of miR-451a expression was observed between A and T groups (*p* = 0.0454), between A and AT groups (*p* = 0.0177), between T and AT groups (*p* = 0.0494). The data was represented as the mean and SE. ER, estrogen receptor; PR, progesterone receptor; HER2, human epidermal growth factor receptor 2; A, anthracycline; T, taxol; AT, anthracycline and taxol combination. SE, standard error.

10 Paraffin embedded sections of 4 μm thick were cut, dewaxed, and lightly stained with hematoxylin. Under a dissecting microscope, tumor tissues were microdissected with a 25 G needle [8]. Then microdissected tissue was dissolved in a storage solution. Then miRNA extraction was performed with a miRNeasy FFPE Kit (Bioteke, China) according to the operation instructions, which enables enrichment and purification of total miRNA. The quality of total miRNA was determined by concentration and purity. 10 ng miRNA was reverse transcribed and a qPCR assay was performed with a 7900HT system (Applied Biosystems, Foster city, CA) using All-In-One™ qRT-PCR detection kit (Genecopeia, Rockville, MD, United States) according to the manufacturer’s instructions. All primers of selected miRNAs and endogenous control U6 were purchased from Genecopeia (Rockville, MD, United States). Relative expression of selected miRNAs was calculated using a traditional formula: 2^−△Ct^ (△Ct = (Ct_miRNA)-(Ct_U6)). All reactions were run in duplicate. The relative expression of each miRNA was represented as the mean and SE (standard error, see Figures 1D–F, 2).

### Molecular Subtypes and Immunohistochemistry Assessment

Invasive breast cancer is divided into four molecular subtypes: Luminal A, Luminal B, HER-2 overexpression and Basic-like types. IHC definition of luminal A tumors was ER/PR positive and HER2 negative with a ≤14% Ki67 index and of luminal B tumors was ER/PR positive and HER2 negative with a >14% Ki67 index or HER2 positive. ER/PR negative and HER-2 positive was included in HER-2 overexpression type. Basal-like types mainly refers to tumors in which the expression of ER、PR and HER-2 are all negative.

ER and PR were defined as positive: 1% tumor cells were nuclear immunostaining. The immunostaining of HER2 was referenced the criterion: 0: No staining or ≤10% of infiltrating cancer cells present incomplete and weak cell membrane staining; 1+: >10% of infiltrating cancer cells present incomplete and weak cell membrane staining; 2+: >10% of infiltrating cancer cells show incomplete and/or weak to moderate cell membrane staining; or ≤10% of infiltrating cancer cells show strong and complete cell membrane staining; 3+: >10% of infiltrating cancer cells show strong and intact cell membrane staining. 0 and 1 + cases were defined as HER-2 negative. 3 + cases were defined as HER-2 positive. All 2 + cases were further performed by FISH to conform HER-2 expression. The cutoff of Ki67 expression was used as 14% [9, 10]. Ki67 low expression was defined as ≤ 14% tumor cells nucleus positive; and >14% tumor cells nucleus positive was defined as Ki67 high expression. Expression of estrogen receptor (ER), progesterone receptor (PR), human epidermal growth factor receptor-2 (HER2), Ki67 and p53 were measured in breast cancer tissues*.* Antibodies against ER, PR, HER-2, Ki67, and p53 (ZA-0102, ZA-0255, ZA-0023, TA500625, ZA-0501; ZSGB-BIO, China) were used for immunohistochemistry. The immunostaining methods was referred to the published article [11]. Immunostaining was confirmed by two pathologists (P Gao and Y-H Li) independently according to the American Society of Clinical Oncology/College of American Pathologists guidelines [12, 13].

### Statistical Analysis

Analyses were performed using statistical software, Prism 5 (GraphPad Software, La Jolla, CA, United States) and SPSS software (version 20.0; Chicago, IL, United States). Differences of miRNAs expression between chemosensitive and chemoresistant groups were compared with the Student *t*-test. And the normality of the data was evaluated before performing the Student *t*-test, based on the following principles: 1) the samples of three sets are large enough (greater than 50), which means approximately to a normal distribution statistically; 2) a scatter plot chart was used to see if sample deviates significantly from the normal distribution. The Chi-square test or Fisher’s exact test was used to analyze the relationship between aberrant miRNA (significantly different (*p* < 0.05) between the chemoresistant and chemosensitive groups) expressions on a dichotomous scale and clinicopathological parameters. Differentially expressed miRNAs of 10 chemosensitive and 10 chemoresistant samples were showed by cluster analysis (using Cluster and TreeView software (http://rana.lbl.gov/EisenSoftware.htm)). The miRNAs with similar expression profiles are enclosed together, and the ruler range shows the Z value range (see Figure 1B). The Z value calculation formula is listed below. Z_sample-i_ = [(log2(Signal_sample-i_)–Mean (Log2(Signal) of all samples)]/[Standard deviation (Log2(Signal) of all samples). To evaluate the predictive value of individual miRNAs and the miRNA signature, receiver operating characteristic (ROC) curves were generated and areas under the curves (AUC) were calculated. The fitted ROC curve is displayed as the true positive rate (TPR) vs. the false positive rate (FPR). AUC, sensitivity and specificity at the optimal cut-off were computed in order to validate the predictive value of each miRNA or miRNA signature on a dichotomous scale. A Logistic regression model (details below: *Results* of Results) was applied to establish a miRNA signature by combining potential biomarker miRNAs [14, 15]. The relationship between the five miRNAs expression and endocrine features or chemotherapy drugs were analyzed by using Student’s *t*-test. *p* values were considered statistically significant at *p* < 0.05.

## Results

### Identification of miRNA Expression Patterns in Chemoresistant and Chemosensitive Breast Cancer Samples

MiRNA microarray analyses of chemoresistant (G1) and chemosensitive (G2) groups showed potential 33 miRNAs to be significantly dysregulated (FC > 1.5 and *p* < 0.05; Figures 1B,C). Compared with the chemosensitive group, nine miRNAs were down-regulated and 24 miRNAs were up-regulated in the chemoresistant group. Excluding those with PCR primers unavailable temporarily for validation (miR-3613-5p、miR-3607-5p、miR-4763、miR-4436b-5p、miR-4638、miR-6503-3p、miR-5588-3p、miR-5193、miR-4731-5p、miR-3126-3p、miR-3607-5p、miR-5095、miR-4758-3p and miR-4279) or with low signal value (miR-4472、miR-1825、miR-1233-1-5p、miR-1234-5p、miR-1268p、miR-142-5p、miR-518f-3p、miR-141-3p、miR-182-5p、miR-103a-3p、miR-24-3p、miR-150-5p and miR-34-3p) in the miRNA microarray, we selected 6 miRNAs: miR-200c-3p, miR-214-3p, miR-23a-3p, and miR-23b-3p, miR-451a and miR-638 (differentially expressed at FC ≥ 1.5 fold [16]) for subsequent validation by qRT-PCR in the training set (*N* = 60). qRT-PCR results confirmed six miRNAs were significantly associated with chemoresistance (*p* < 0.05; FC > 1.5), which conclusion was the same as the microarray data. Of these six miRNAs, expression of miR-200c-3p, miR-214-3p, miR-23a-3p, and miR-23b-3p were positively associated with chemoresistance, and expression of miR-451a and miR-638 were negatively associated with chemoresistance (Figure 1D). To further validate these data, qRT-PCR was performed on six miRNAs in the internal testing set (*N* = 59) and the independent (*N* = 71) set. Expression of these six miRNAs was significantly different between chemoresistant and chemosensitive groups in the internal testing set (Figure 1E), in the independent set (Figure 1F) and in combined set (including the training, internal testing and independent set; Figure 1G).

### Candidate Biomarker miRNAs Were Selected by ROCs

**FIGURE 3:**
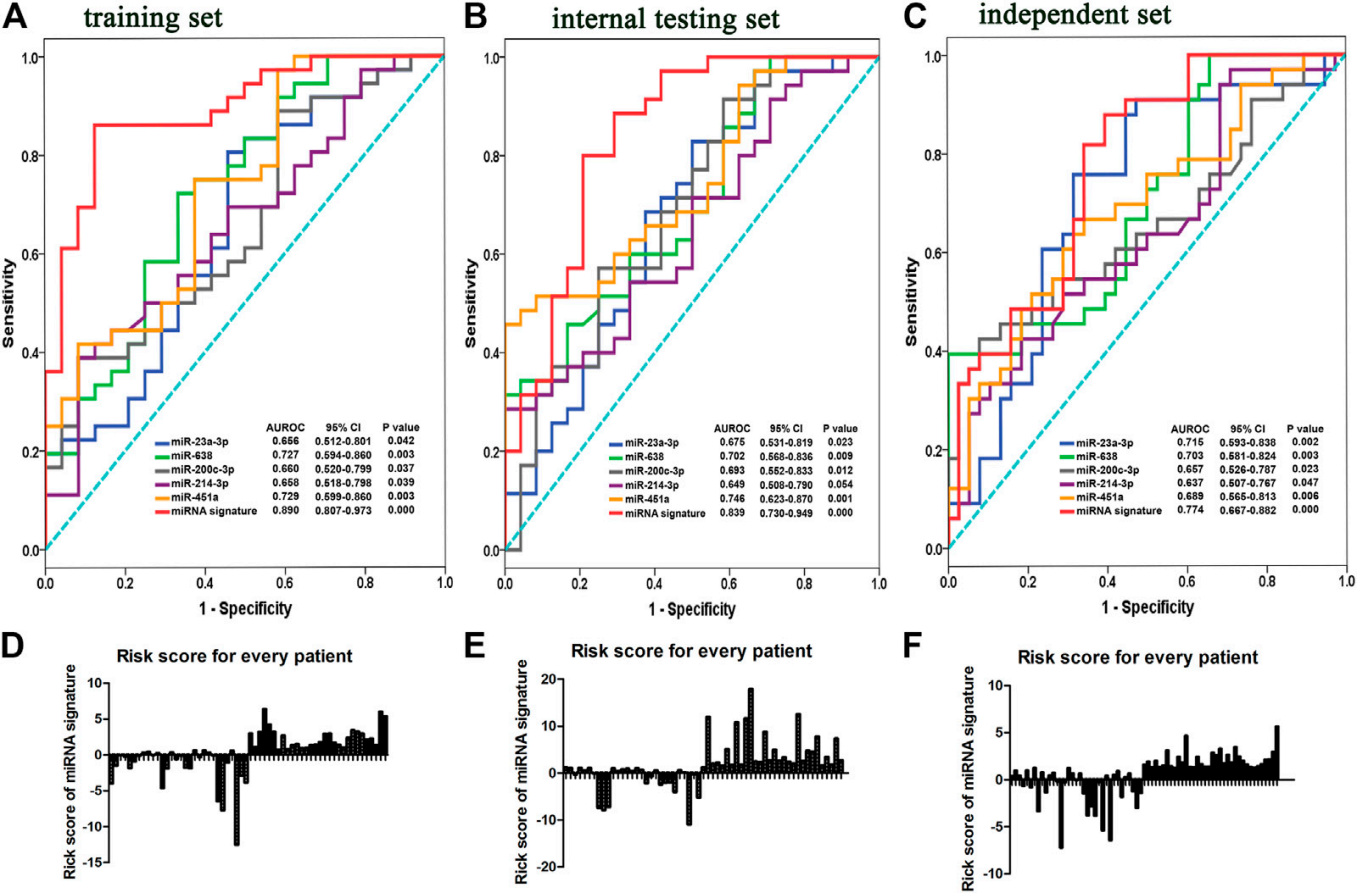
Risk scores according to five-miRNA signatures and ROC curves in training, internal testing and independent sets. ROC and AUCs of single miRNA alone and five-miRNA signature from the training **(A)**, internal testing **(B)**, and independent sets **(C)**. Risk scores for patients were calculated by risk scores based on a five-miRNA signature in the training **(D)**, internal testing. **(E)** and independent sets **(F)**.

The expression of each miRNA was divided into high or low level with the median as a cut-off point [17, 18]. According to this cut-off value, six miRNAs were divided into high-expressed or low-expressed group. Then the predictive accuracy of one single miRNA to distinguish between chemosensitive and chemoresistant breast cancer was assessed by a ROC test in the training set. Considered individually, ROC curve showed AUC of miR-23b-3p was lower (AUC = 0.638; 95%CI: 0.385-0.685; Supplementary Figure S1) and *p* value was not reached statistical significance (*p* = 0.073). Thus, miR-23b-3p was eliminated from the candidate biomarkers. miR-23a-3p (AUC = 0.656; 95% CI: 0.512–0.801; *p* = 0.042), miR-638 (AUC = 0.727; 95% CI: 0.594–0.860; *p* = 0.003), miR-200c-3p (AUC = 0.660; 95% CI: 0.520–0.799; *p* = 0.037), miR-214–3p (AUC = 0.658; 95% CI: 0.518–0.798; *p* = 0.039), and miR-451a (AUC = 0.729; 95% CI: 0.599–0.860; *p* = 0.003) had significantly higher AUC scores (Figure 3A; all *p* < 0.05). To validate the five potential miRNAs, ROCs for internal testing and independent sets were also measured (Figures 3B,C). Data show that all the five miRNAs had stable predictive value for chemotherapeutic response (Supplementary Figure S2). The relationship between expression of the five miRNAs with clinical characteristics of training, internal testing and independent sets appears in Supplementary Tables S1–S5.

### Five-miRNA Signatures Were Generated by Logistic Regression

To evaluate the value of miRNAs as a biomarker, we integrated the above five miRNAs (miR-23a-3p, miR-638, miR-200c-3p, miR-214-3p, and miR-451a) to build a predictive classifier in the training set. miRNAs were analyzed by resorting to a Logistic regression model on their continuous original scale. Chemosensitivity (defined as “0”) or chemoresistance (defined as “1”) is regarded as an event. By SPSS software, the contribution value (a regression coefficient) to chemotherapy resistance of each miRNA was calculated and used as its weight. We obtained a formula to calculate the risk score for every patient, weighted by a regression coefficient and added a balance count: Risk score = (0.782 × expression of miR-23a-3p) − (3.968 × expression of miR-638) + (0.361 × expression of miR-200c-3p) + (5.848 × expression of miR-214-3p) − (2.598 × expression of miR-451a) + 0.562.

With the risk score formula, we calculated a risk score for each patient from the three sets based on individual expression of the five miRNAs (Figures 3D–F). Patients in the training set were divided into low- and high-risk groups, with the median risk score as a threshold. Compared with low-risk scoring patients, high-risk scoring patients tended to be resistant to chemotherapy (AUC = 0.890; 95% CI: 0.807–0.973; *p* = 0.000, Figure 3A). Patients in the internal testing set were classified into low- and high-risk groups with the same formula as that used in the training set. As expected, patients in the validation set with high-risk scores had resistant chemotherapeutic responses (AUC = 0.839; 95% CI: 0.730–0.949; *p* = 0.000; Figure 3B). To assess whether the miRNA signature had the same or similar predictive value in different populations, patients in the independent set were divided into low- and high-risk groups and similar data were noted (AUC = 0.774; 95% CI: 0.667–0.882; *p* = 0.000; Figure 3C). The five-miRNA signature had better predictive accuracy (higher AUC, *p* < 0.05) than each single miRNA for distinguishing breast cancer patients with a chemoresistant response from those who were chemosensitive. Detailed clinical characteristics of patients according to the microRNA signature in training, internal testing, and independent set was showed in Table 2.

**TABLE 2:** Clinical characteristics of patients according to the microRNA signature in training, internal testing, and independent set.

	Training set (*N* = 60)			Internal testing set (*N* = 59)			Independent set (*N* = 71)		
	Low-risk	High-risk	*p* Value	Low-risk	High-risk	*p* Value	Low-risk	High-risk	*p* Value
	Group (*n* = 30)	Group (*n* = 30)		Group (*n* = 29)	Group (*n* = 30)		Group (*n* = 35)	Group (*n* = 36)	
Age									
≤40	4	5		9	7		8	8	
41–60	20	19		15	21		20	23	
≥61	6	6	1.0000	5	2	0.9609	7	5	0.9641
Lymph node metastasis									
absent	7	7		10	4		8	16	
present	23	23	1.0000	19	26	0.0716	27	20	0.0791
T Stage									
T1	5	6		11	6		5	5	
T2	21	21		12	19		25	20	
T3	4	3	0.8897	6	5	0.2095	5	11	0.2476
*N* stage									
N0	7	7		10	4		13	11	
N1	9	7		5	3		9	8	
N2	9	8		8	13		7	9	
N3	5	8	0.8010	6	10	0.1546	6	8	0.8620
TNM stage									
I	1	2		3	0		3	3	
II	12	9		10	7		15	14	
III	16	16		15	23		17	19	
IV	1	3	0.6233	1	0	0.1023	0	0	0.9363
Chemotherapy drug									
Doxorubicin	5	6		1	3		2	1	
Taxol	6	12		8	5		0	2	
Doxoribicin + Taxol	18	11		20	19		30	33	
others	1	1	0.2682	0	3	0.1949	3	0	0.1409
Chemotherapy cycles									
≤4	22	23		15	23		21	22	
>4	8	7	1.0000	14	7	0.0596	14	14	1.0000
Chemotherapy assessment									
resistance	9	27		10	25		10	23	
sense	21	3	<0.0001^*^	19	5	0.0002^*^	25	13	0.0042^*^
Histological grade									
I	3	1		0	1		3	4	
II	11	17		23	21		18	15	
III	16	12	0.2397	6	8	0.5066	14	17	0.7075
ER									
negative	8	9		9	9		17	15	
positive	22	21	1.0000	20	21	1.0000	18	21	0.6365
PR									
negative	13	12		12	12		26	21	
positive	17	18	1.0000	17	18	1.0000	9	15	0.2109
HER2									
negative	15	16		6	11		8	8	
positive	15	14	1.0000	23	19	0.2516	27	28	1.0000
P53									
negative	17	7		12	8		11	8	
positive	13	23	0.0169^*^	17	22	0.2789	24	28	0.4304
Ki-67									
≤14%	9	7		6	7		14	8	
>14%	21	23	0.7710	23	23	1.0000	21	28	0.1285
Molecular subtype									
HER2-overexpress	5	5		8	7		12	12	
Basal-like	3	4		1	2		4	3	
Luminal A	12	12		5	9		4	6	
Luminal B	10	9	0.9783	15	12	0.6020	15	15	0.9125

AC: Anthracycline and Cyclophosphamide; CTF: Cyclophosphamide, Taxol and Fluorouracil; AT: Anthracycline and Taxol; CMF: Cyclophosphamide, Methotrexate and Fluorouracil. *Significance different.

### Association Between Five Deregulated miRNAs and Endocrine Features or Chemotherapy Drugs

To understand the role of these five miRNAs in breast cancer chemoresistance, relative expression of the five miRNAs with endocrine features were analyzed in a combined training and internal testing set (*N* = 119). Interestingly, compared with ER-positive cases, miR-451a was under-expressed in cases with ER-negative expression (*p* = 0.0296; Figure 2A). miR-23a expression was higher in PR-positive cases compared to PR-negative cases (*p* = 0.0367; Figure 2B). miR-451a and miR-638 expression were both reduced in HER2-negative cases, compared with HER2-positive cases (*p* = 0.0219, 0.0117; Figures 2C,D). Higher expression of miR-638 and miR-200c-3p was observed in Ki67-high cases compared to Ki67-low cases (*p* = 0.0117, 0.0241; Figures 2E,F). miR-214-3p expression was increased in p53-positive cases compared with p53-negative cases (*p* = 0.0213; Figure 2G). Thus, the five miRNAs had different roles in the development of different breast cancer phenotypes.

To explore whether the five miRNAs mediated chemoresistance associated with different chemotherapy drugs, chemotherapy drug of patients were also collected. The patients used with Anthracycline and Cyclophosphamide (AC) chemotherapy, were incorporated in Anthracycline (A) group. The patients used with Cyclophosphamide, Taxol and Fluorouracil (CTF) chemotherapy, were included in Taxol (T) group. And the patients used with anthracycline and Taxol combined (AT) chemotherapy were absorbed in AT group (AT). The patients in other group were used with Cyclophosphamide, Methotrexate and Fluorouracil (CMF) combined chemotherapy. We compared expression of the five miRNAs among the three groups: A, T and AT groups. Figure 2H shows that miR-23a-3p expression in A and T groups were higher than that of the AT group (*p* = 0.0393, 0.0430). Compared with A and AT groups, miR-200c-3p expression was downregulated in the T group (*p* = 0.0177, 0.0361; Figure 2I). The least expression of miR-638 occurred in the A group, lower than T and AT groups (*p* = 0.019, 0.0162; Figure 2J). Greatest expression of miR-214-3p was observed in T group, higher than A and AT groups (*p* = 0.0254, 0.0373; Figure 2K). A stepwise decrease in miR-451a expression was found in all three groups (Figure 2L). Significant difference of miR-451a expression was observed between A and T groups (*p* = 0.0454), between A and AT groups (*p* = 0.0177), between T and AT groups (*p* = 0.0494). Thus, cases with downregulated expression of miR-638 and miR-451a may be resistant to anthracycline; whereas, cases with upregulated expression of miR-214-3p may be resistant to taxol. Patients with high miR-200c-3p and miR-23a-3p expression could be sensitive to taxol or a combination of anthracycline and taxol.

### Identification of Canonical Pathways by Which the Five miRNAs Modulate Drug Resistance

**FIGURE 4:**
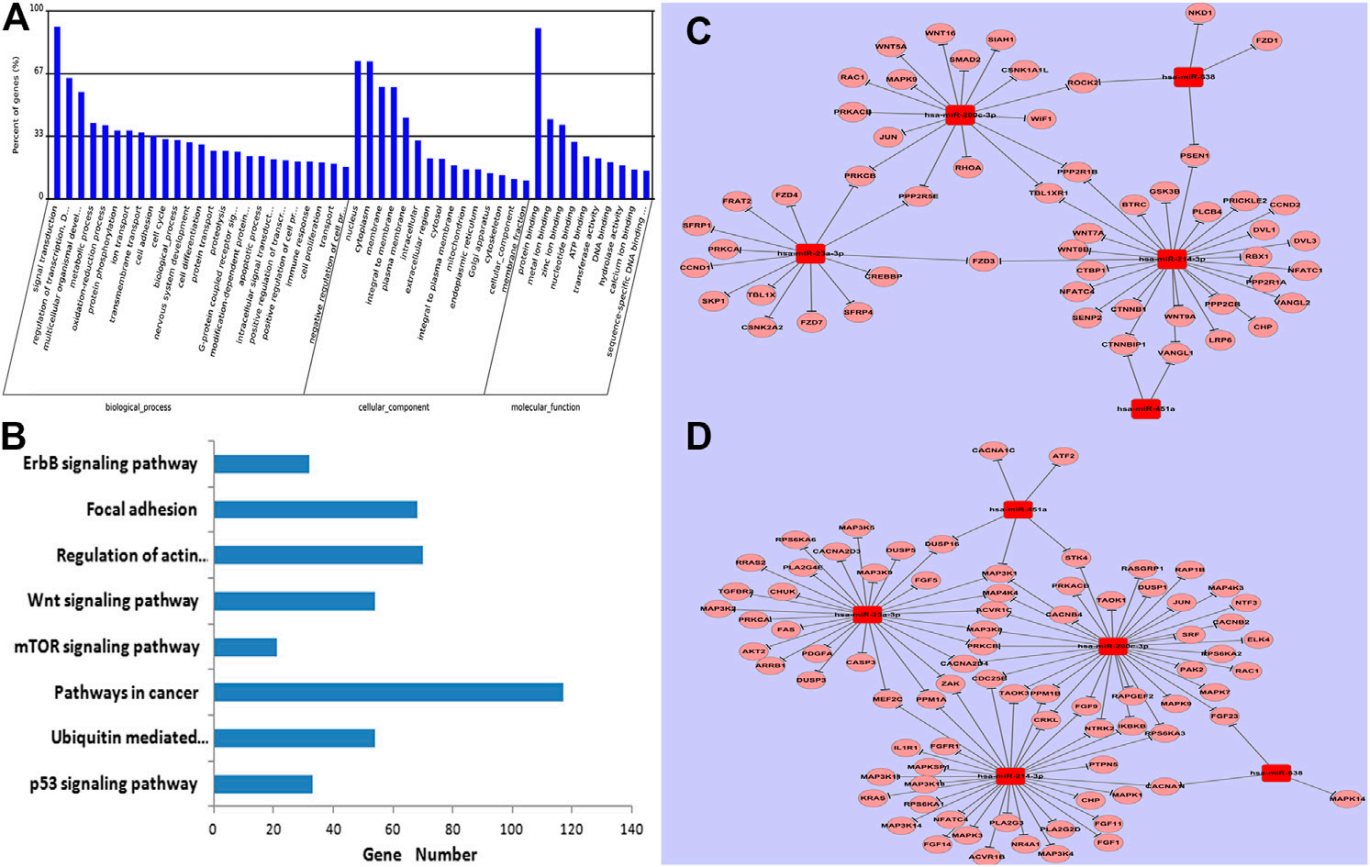
GO and KEGG analysis of predicted targets of five miRNAs. Data show that genes were enriched in biological processes of signal transduction. **(A)**; eight known canonical cancer-associated pathways were predicted by KEGG. **(B)**, including the p53 signaling pathway, ubiquitin mediated proteolysis, pathways in cancer, the mTOR signaling pathway, the Wnt signaling pathway, regulation of actin cytoskeleton, focal adhesion, and the ErbB signaling pathway. Networks among specific deregulated miRNAs and predicted targets of the classical Wnt signaling pathway. **(C)** and the MAPK signaling pathway **(D)**.

To explore potential roles of these five differentially expressed miRNAs in drug-resistance of breast cancer, putative gene targets of the five miRNAs were predicted using three algorithms, including Targetscan, miRanda and PICTA. Targets of miRNAs are mainly involved in signal transduction according to Gene Ontology (GO) enrichment analysis (Figure 4A). So, 1,324 significant target genes of the miRNAs resided within 21 biological pathways according to KEGG analysis, including seven known canonical cancer-associated pathways (Figure 4B): the p53 signaling pathway, ubiquitin mediated proteolysis, pathways in cancer, the mTOR signaling pathway, the Wnt signaling pathway, regulation of actin cytoskeleton, focal adhesion, and the ErbB signaling pathway. Of note, 117 genes were involved in cancer pathways, which suggested that they may be important to breast cancer carcinogenesis. Another 54 target genes were predicted to participate in the classical Wnt signaling pathway (Figure 4C). Additionally, 81 target genes were found to be distributed in the MAPK signaling pathway, which is an important pathway in the breast cancer drug-resistance process (Figure 4D; *p* > 0.05).

## Discussion

Chemotherapy is essential for breast cancer treatment. The patients can be frequently chemoresistant and this causes worse prognosis and greater risk of metastasis and recurrence. This, we must identify specific biomarkers for drug-resistant breast cancer. We firstly established and validated a novel signature based on five-miRNA to predict chemotherapeutic response for breast cancer patients. ROC analysis suggested that a five-miRNA signature was better for predicting chemosensitivity/resistance compared to a single miRNA. In our model, patients with high-risk scores were prone to resist chemotherapy. This signature may help identify patients who would best benefit from chemotherapy and to improve personalized treatment.

We assessed 190 cases of FFPE needle biopsy tissues of breast cancer to analyze miRNAs differential expression. miRNAs maintain stability in FFPE tissues and an abundance of information about cancer behavior and patient outcome made this convenient for clinical application [19, 20]. Gene expression profiling, such as studying 21 chemoresistance-related genes in breast cancer, may help predict clinical outcomes and chemotherapeutic response [21]. However, gene profiling is not economical and most gene profiling is limited to fresh paraffin-embedded or frozen tissues. miRNA microarray is almost not affected by DNA degradation, and many genes are regulated by miRNAs, information obtained from miRNA profiling may be more thorough and accurate than gene chip expression assessment. To our knowledge, ours is the first to demonstrate that a five-miRNA signature of breast cancer prior to NAC may predict response to chemotherapy.

Previous studies indicate that multiple miRNAs exert crucial functions in drug resistance of human cancers, including the five miRNAs used in our classifier. For example, miR-451a was reported to target proliferative and anti-apoptotic factor 14-3-3ξ and mediate tamoxifen resistance of breast cancer [22]. Yu and colleagues’ compared cisplatin-sensitive and resistant cell lines in tongue squamous cell carcinoma with miRNA microarray and reported that miR-214 served as a chemoresistant miRNA, as well as miR-23a [23]. Increased miR-638 expression was observed in cisplatin-induced apoptosis in SPC-A1 cells of NSCLC [24]. qRT-PCR analysis by Lv’s group confirmed that compared to a parental MCF-7 cell line, miR-200c was over-expressed in MCF-7/ADM cells, suggesting a role in doxorubicin resistance [25].

Endocrine therapy is also used to treat breast cancer. The association between the five deregulated miRNAs and ER or PR was analyzed in our study. Compared with ER-positive cases, miR-451a expression was decreased in ER-negative cases. miR-23a-3p expression was increased in PR-negative cases compared with PR-positive ones. Thus, endocrine therapy may not help patients with low miR-451a or high miR-23a-3p expression. Molecular targeted therapy, such as trastuzumab [26] has been widely used for breast cancer treatment. miR-638 and miR-451a expression were downregulated in HER2 -negative breast cancer samples compared to those with HER2-positive expression. So we speculated that molecular targeted drugs against HER2 might not be effective for patients with low expression of miR-638 or miR-451a. Additionally, cell proliferation and apoptosis was closely correlated with drug resistance. High miR-200c-3p and miR-214-3p expression was observed in Ki67-positive and p53-negative breast cancer cases, which suggest that both miRNAs might participate in chemoresistance by promoting cell proliferation or repressing apoptosis.

Furthermore, the drug-resistant mechanism of miRNAs might be related to different chemotherapy drugs. From 2019 to 2013, neoadjuvant chemotherapy of this study mainly includes four types: Anthracycline and Cyclophosphamide (AC) chemotherapy; Cyclophosphamide, Taxol and Fluorouracil (CTF) chemotherapy, anthracycline and Taxol combined (AT) chemotherapy and Cyclophosphamide, Methotrexate and Fluorouracil (CMF) chemotherapy. The main mechanism of resistance is related to Anthracyclines and Taxol drugs. Our data also suggest that restoration expression of miR-451a and miR-638 may enhance chemosensitivity to anthracycline. Taxol may be effective for patients with high miR-200c-3p expression. In contrast, patients with high miR-214c-3p expression may be taxol-resistant. Combination therapy of anthracycline and taxol may be useful for patients with high miR-23a-3p expression.

Our study was limited by different collection sites for samples [training and internal testing sets were collected from Qilu Hospital, Shandong University (Jinan, China); an independent set was collected from Liaocheng People’s Hospital (Liaocheng, China)]. Also, we may not be able to generalize our data to other nationalities as all our samples were Chinese. Thus, specific clinical characteristics may be attributed to ethnicity and this miRNA signature may be inapplicable to broader populations. Larger studies with more diverse samples would be helpful for confirming our data.

In summary, we investigated the potential predictive value of miRNAs in drug-resistant breast cancer and we found that integration of five miRNAs as one “tool” had greater accuracy and predictive value for chemotherapeutic response. Using this innovative miRNA signature, which might be helpful for personalized chemotherapeutic regimens of breast cancer patients, and it will protect resistant patients from ineffective treatments.

## Data Availability

The datasets presented in this study can be found in online repositories. The names of the repository/repositories and accession numbers can be found in the article/Supplementary Material.
