# Supplementary material for: Identification of miRNA Signature in Breast Cancer to Predict Neoadjuvant Chemotherapy Response
Source: Pathol Oncol Res. 2021 Apr 30;27:1609753. doi: 10.3389/pore.2021.1609753 (PMC8262148; doi:10.3389/pore.2021.1609753)
Supplement: Supplementary file 1 [file Table1.DOC]

**Fig. S1**


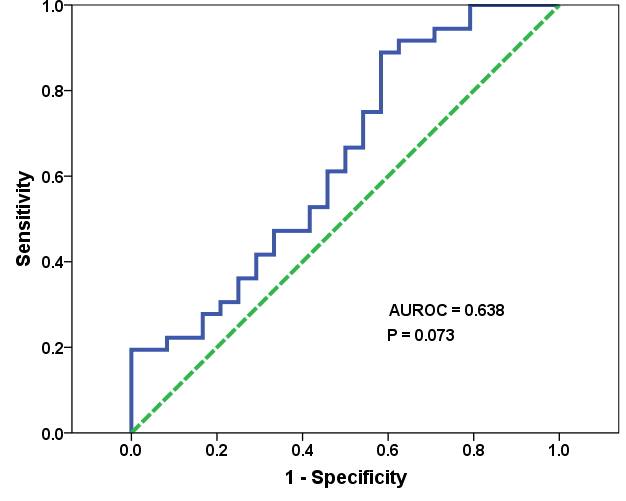


The predictive value of miR-23b-3p in chemoresistance was tested by ROC curves. The results showed AUC score was 0.638; however, it was not statistically significant (*P* > 0.05).

**Fig. S2**

**
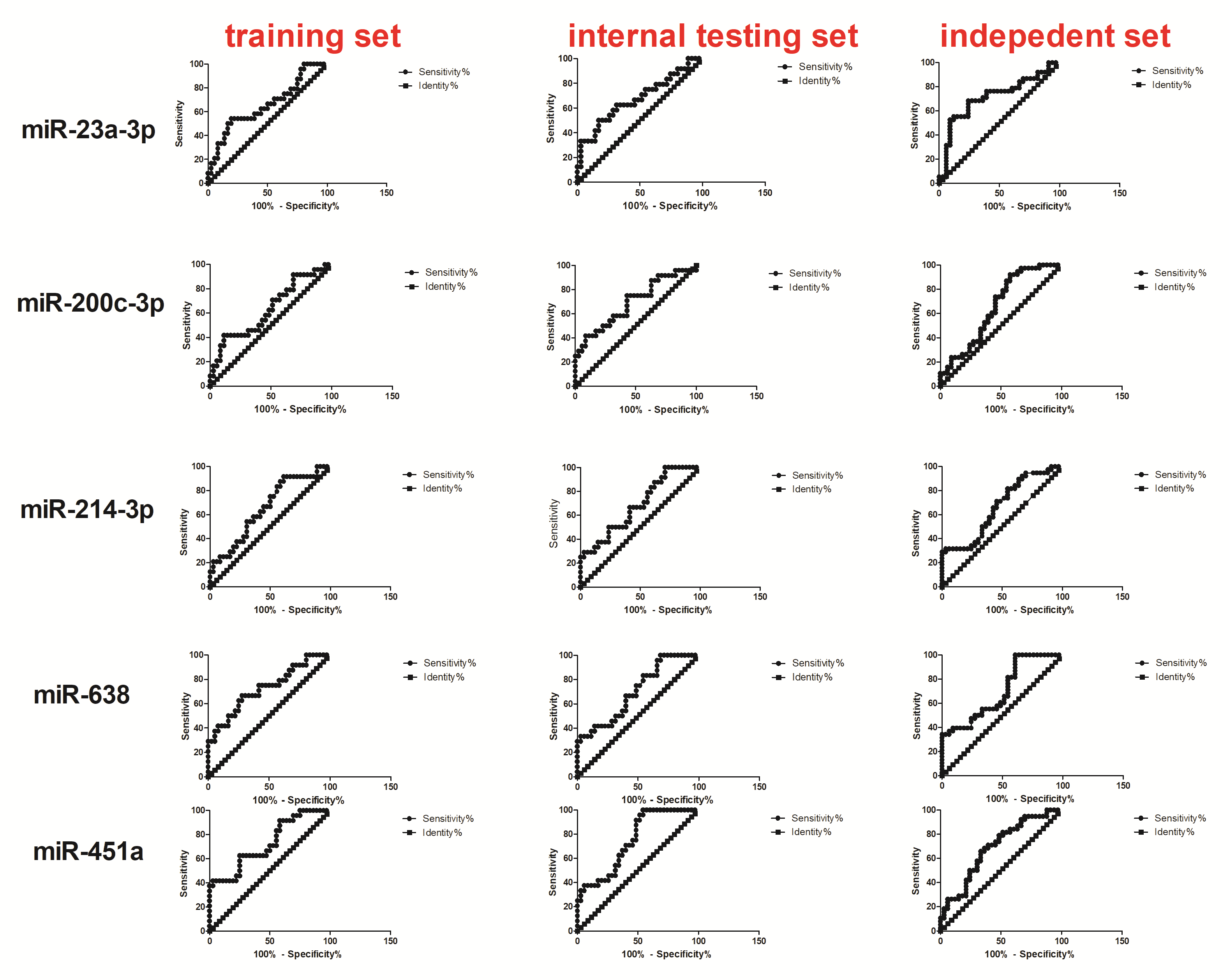
**

miR-23a-3p, miR-200c-3p, miR-214-3p, miR-638 and miR-451a had stable predictive value for chemotherapeutic response in training set (AUC=0.656; 0.660; 0.658; 0.727; 0.729), internal testing set (AUC=0.675; 0.693; 0.649; 0.702; 0.746) and independent set (AUC=0.715; 0.657; 0.637; 0.703; 0.689; all *P* < 0.05).

**Table S1:**

**Association of clinical characteristics of patients** with miR-23a-3p expression

|  | Training set (N=60) | | | Internal testing set (N=59) | | | Independent set (N=71) | | |
| --- | --- | --- | --- | --- | --- | --- | --- | --- | --- |
|  | Low-level (n=30) | High-level  (n=30) | *P* value | Low-level  (n=29) | High-level  (n=30) | *P* value | Low-level  (n=35) | High-level  (n=36) | *P* value |
| Age |  |  |  |  |  |  |  |  |  |
| ≤40  41-60 | 6  17 | 3  22 |  | 3  21 | 13  15 |  | 6  21 | 10  22 |  |
| ≥61 | 7 | 5 | 0.3726 | 5 | 2 | 0.0141* | 8 | 4 | 0.3099 |
| Lymph node  metastasis |  |  |  |  |  |  |  |  |  |
| absent | 5 | 9 |  | 8 | 6 |  | 15 | 9 |  |
| present | 25 | 21 | 0.3604 | 21 | 24 | 0.5520 | 20 | 27 | 0.1368 |
| T stage |  |  |  |  |  |  |  |  |  |
| T1 | 6 | 5 |  | 8 | 9 |  | 4 | 6 |  |
| T2 | 19 | 23 |  | 16 | 15 |  | 25 | 20 |  |
| T3 | 5 | 2 | 0.4153 | 5 | 6 | 0.9208 | 6 | 10 | 0.3787 |
| N stage |  |  |  |  |  |  |  |  |  |
| N0 | 5 | 9 |  | 8 | 6 |  | 15 | 9 |  |
| N1 | 10 | 6 |  | 4 | 4 |  | 5 | 12 |  |
| N2 | 7 | 10 |  | 12 | 9 |  | 8 | 8 |  |
| N3 | 8 | 5 | 0.3388 | 5 | 11 | 0.3997 | 7 | 7 | 0.2243 |
| TNM stage |  |  |  |  |  |  |  |  |  |
| I | 1 | 2 |  | 2 | 1 |  | 3 | 3 |  |
| II | 10 | 11 |  | 9 | 8 |  | 15 | 14 |  |
| III | 17 | 15 |  | 18 | 20 |  | 17 | 19 |  |
| IV | 2 | 2 | 0.9176 | 0 | 1 | 0.6867 |  |  | 0.9363 |
| Chemotherapy drug |  |  |  |  |  |  |  |  |  |
| AC | 4 | 7 |  | 3 | 1 |  | 2 | 1 |  |
| CTF | 7 | 11 |  | 6 | 7 |  | 0 | 2 |  |
| AT | 17 | 12 |  | 20 | 19 |  | 31 | 32 |  |
| CMF | 2 | 0 | 0.2062 | 0 | 3 | 0.2522 | 2 | 1 | 0.4455 |
| Chemotherapy  cycles |  |  |  |  |  |  |  |  |  |
| ≤4 | 19 | 26 |  | 18 | 20 |  | 21 | 22 |  |
| >4 | 11 | 4 | 0.0716 | 11 | 10 | 0.7892 | 14 | 14 | 1.0000 |
| Chemotherapy assessment |  |  |  |  |  |  |  |  |  |
| resistance | 13 | 23 |  | 13 | 22 |  | 9 | 24 |  |
| sense | 17 | 7 | 0.0169* | 16 | 8 | 0.0352* | 26 | 12 | 0.0008* |
| Histological grade |  |  |  |  |  |  |  |  |  |
| I | 3 | 1 |  | 1 | 0 |  | 3 | 4 |  |
| II | 9 | 19 |  | 18 | 26 |  | 18 | 15 |  |
| III | 18 | 10 | 0.0324* | 10 | 4 | 0.0817 | 14 | 17 | 0.7075 |
| ER |  |  |  |  |  |  |  |  |  |
| negative | 10 | 7 |  | 9 | 9 |  | 12 | 20 |  |
| positive | 20 | 23 | 0.5675 | 20 | 21 | 1.0000 | 23 | 16 | 0.0962 |
| PR |  |  |  |  |  |  |  |  |  |
| negative | 15 | 10 |  | 13 | 11 |  | 23 | 24 |  |
| positive | 15 | 20 | 0.2949 | 16 | 19 | 0.6010 | 12 | 12 | 1.0000 |
| HER2 |  |  |  |  |  |  |  |  |  |
| negative | 15 | 16 |  | 9 | 8 |  | 7 | 9 |  |
| positive | 15 | 14 | 1.0000 | 20 | 22 | 0.7787 | 28 | 27 | 0.7775 |
| P53 |  |  |  |  |  |  |  |  |  |
| negative | 11 | 13 |  | 12 | 8 |  | 9 | 10 |  |
| positive | 19 | 17 | 0.7925 | 17 | 22 | 0.2789 | 26 | 26 | 1.0000 |
| Ki-67 |  |  |  |  |  |  |  |  |  |
| ≤14% | 9 | 7 |  | 7 | 6 |  | 12 | 10 |  |
| >14% | 21 | 23 | 0.7710 | 22 | 24 | 1.0000 | 23 | 26 | 0.6138 |
| Molecular subtype |  |  |  |  |  |  |  |  |  |
| HER2-overexpress | 5 | 5 |  | 8 | 7 |  | 10 | 14 |  |
| Basal-like | 3 | 4 |  | 1 | 2 |  | 1 | 6 |  |
| Luminal A | 12 | 12 |  | 8 | 6 |  | 6 | 4 |  |
| Luminal B | 10 | 9 | 0.9783 | 12 | 15 | 0.8007 | 18 | 12 | 0.1204 |

*Significance different AC: Anthracycline and Cyclophosphamide; CTF: Cyclophosphamide, Taxol and Fluorouracil; AT: Anthracycline and Taxol; CMF: Cyclophosphamide, Methotrexate and Fluorouracil.

**Table S2:**

**Association of clinical characteristics of patients with miR-638** expression

|  | Training set (N=60) | | | Internal testing set (N=59) | | | Independent set (N=71) | | |
| --- | --- | --- | --- | --- | --- | --- | --- | --- | --- |
|  | Low-level (n=30) | High-level  (n=30) | *P* value | Low-level  (n=29) | High-level  (n=30) | *P* value | Low-level  (n=35) | High-level  (n=36) | *P* value |
| Age |  |  |  |  |  |  |  |  |  |
| ≤40  41-60 | 3  24 | 6  15 |  | 8  15 | 8  22 |  | 7  20 | 9  23 |  |
| ≥61 | 3 | 9 | 0.0479* | 6 | 1 | 0.0892 | 8 | 4 | 0.4109 |
| Lymph node  metastasis |  |  |  |  |  |  |  |  |  |
| absent | 5 | 9 |  | 5 | 9 |  | 14 | 10 |  |
| present | 25 | 21 | 0.3604 | 24 | 21 | 0.9685 | 21 | 26 | 0.9595 |
| T stage |  |  |  |  |  |  |  |  |  |
| T1 | 4 | 7 |  | 7 | 10 |  | 3 | 7 |  |
| T2 | 23 | 19 |  | 17 | 14 |  | 22 | 23 |  |
| T3 | 3 | 4 | 0.5112 | 5 | 6 | 0.6396 | 10 | 6 | 0.2714 |
| pN stage |  |  |  |  |  |  |  |  |  |
| N0 | 5 | 9 |  | 5 | 9 |  | 14 | 10 |  |
| N1 | 9 | 7 |  | 2 | 6 |  | 8 | 12 |  |
| N2 | 10 | 7 |  | 16 | 5 |  | 7 | 6 |  |
| N3 | 6 | 7 | 0.5726 | 6 | 10 | 0.0195* | 6 | 8 | 0.6829 |
| TNM stage |  |  |  |  |  |  |  |  |  |
| I | 1 | 2 |  | 0 | 3 |  | 2 | 4 |  |
| II | 10 | 11 |  | 7 | 10 |  | 14 | 15 |  |
| III | 16 | 16 |  | 22 | 16 |  | 19 | 17 |  |
| IV | 3 | 1 | 0.7100 | 0 | 1 | 0.1410 |  |  | 0.6709 |
| Chemotherapy drug |  |  |  |  |  |  |  |  |  |
| AC | 4 | 7 |  | 3 | 1 |  | 1 | 2 |  |
| CTF | 11 | 7 |  | 6 | 7 |  | 1 | 1 |  |
| AT | 15 | 14 |  | 17 | 22 |  | 33 | 30 |  |
| CMF | 0 | 2 | 0.2908 | 3 | 0 | 0.1949 | 0 | 3 | 0.3256 |
| Chemotherapy  cycles |  |  |  |  |  |  |  |  |  |
| ≤4 | 23 | 22 |  | 21 | 17 |  | 23 | 20 |  |
| >4 | 7 | 8 | 1.0000 | 8 | 13 | 0.2789 | 12 | 16 | 0.4687 |
| Chemotherapy assessment |  |  |  |  |  |  |  |  |  |
| resistance | 24 | 12 |  | 13 | 22 |  | 19 | 14 |  |
| sense | 6 | 18 | 0.0033* | 16 | 8 | 0.0159* | 16 | 22 | 0.0329* |
| Histological grade |  |  |  |  |  |  |  |  |  |
| I | 3 | 1 |  | 1 | 0 |  | 4 | 3 |  |
| II | 16 | 12 |  | 20 | 24 |  | 17 | 16 |  |
| III | 11 | 17 | 0.2397 | 8 | 6 | 0.4420 | 14 | 17 | 0.7987 |
| ER |  |  |  |  |  |  |  |  |  |
| negative | 9 | 8 |  | 7 | 11 |  | 16 | 16 |  |
| positive | 21 | 22 | 1.0000 | 22 | 19 | 0.3895 | 19 | 20 | 1.0000 |
| PR |  |  |  |  |  |  |  |  |  |
| negative | 13 | 12 |  | 10 | 14 |  | 22 | 25 |  |
| positive | 17 | 18 | 1.0000 | 19 | 16 | 0.4296 | 13 | 11 | 0.6210 |
| HER2 |  |  |  |  |  |  |  |  |  |
| negative | 17 | 14 |  | 10 | 7 |  | 7 | 9 |  |
| positive | 13 | 16 | 0.6058 | 19 | 23 | 0.3985 | 28 | 27 | 0.7775 |
| P53 |  |  |  |  |  |  |  |  |  |
| negative | 10 | 14 |  | 11 | 9 |  | 7 | 12 |  |
| positive | 20 | 16 | 0.4296 | 18 | 21 | 0.5889 | 28 | 23 | 0.2848 |
| Ki-67 |  |  |  |  |  |  |  |  |  |
| ≤14% | 4 | 12 |  | 7 | 6 |  | 9 | 13 |  |
| >14% | 26 | 18 | 0.0391* | 22 | 24 | 1.0000 | 26 | 23 | 0.4432 |
| Molecular subtype |  |  |  |  |  |  |  |  |  |
| HER2-overexpress | 6 | 4 |  | 6 | 9 |  | 13 | 11 |  |
| Basal-like | 2 | 5 |  | 1 | 2 |  | 2 | 5 |  |
| Luminal A | 15 | 9 |  | 9 | 5 |  | 5 | 5 |  |
| Luminal B | 7 | 12 | 0.2122 | 13 | 14 | 0.5525 | 15 | 15 | 0.6965 |

*Significance different AC: Anthracycline and Cyclophosphamide; CTF: Cyclophosphamide, Taxol and Fluorouracil; AT: Anthracycline and Taxol; CMF: Cyclophosphamide, Methotrexate and Fluorouracil.

**Table S3:**

**Association of clinical characteristics of patients with miR-200c-3p** expression

|  | Training set (N=60) | | | Internal testing set (N=59) | | | Independent set (N=71) | | |
| --- | --- | --- | --- | --- | --- | --- | --- | --- | --- |
|  | Low-level (n=30) | High-level  (n=30) | *P* value | Low-level  (n=29) | High-level  (n=30) | *P* value | Low-level  (n=35) | High-level  (n=36) | *P* value |
| Age |  |  |  |  |  |  |  |  |  |
| ≤40  41-60 | 3  19 | 6  20 |  | 4  21 | 12  15 |  | 11  19 | 5  24 |  |
| ≥61 | 8 | 4 | 0.3074 | 4 | 3 | 0.0770 | 5 | 7 | 0.2069 |
| Lymph node  metastasis |  |  |  |  |  |  |  |  |  |
| absent | 6 | 8 |  | 9 | 5 |  | 13 | 11 |  |
| present | 24 | 22 | 0.7611 | 20 | 25 | 0.2326 | 22 | 25 | 0.6210 |
| T stage |  |  |  |  |  |  |  |  |  |
| T1 | 2 | 9 |  | 7 | 10 |  | 8 | 2 |  |
| T2 | 24 | 18 |  | 14 | 17 |  | 23 | 22 |  |
| T3 | 4 | 3 | 0.0654 | 8 | 3 | 0.2148 | 4 | 12 | 0.0223* |
| N stage |  |  |  |  |  |  |  |  |  |
| N0 | 6 | 8 |  | 9 | 5 |  | 13 | 11 |  |
| N1 | 8 | 8 |  | 4 | 4 |  | 7 | 10 |  |
| N2 | 7 | 10 |  | 9 | 12 |  | 9 | 7 |  |
| N3 | 9 | 4 | 0.4338 | 7 | 9 | 0.6138 | 6 | 8 | 0.7487 |
| TNM stage |  |  |  |  |  |  |  |  |  |
| I | 0 | 3 |  | 1 | 2 |  | 5 | 1 |  |
| II | 10 | 11 |  | 11 | 6 |  | 13 | 16 |  |
| III | 18 | 14 |  | 17 | 21 |  | 17 | 19 |  |
| IV | 2 | 2 | 0.3146 | 0 | 1 | 0.3605 |  |  | 0.2150 |
| Chemotherapy drug |  |  |  |  |  |  |  |  |  |
| AC | 4 | 7 |  | 2 | 2 |  | 1 | 2 |  |
| CTF | 10 | 8 |  | 5 | 8 |  | 2 | 0 |  |
| AT | 14 | 15 |  | 21 | 18 |  | 30 | 33 |  |
| CMF | 2 | 0 | 0.3802 | 1 | 2 | 0.7435 | 2 | 1 | 0.4542 |
| Chemotherapy  cycles |  |  |  |  |  |  |  |  |  |
| ≤4 | 23 | 22 |  | 21 | 17 |  | 20 | 23 |  |
| >4 | 7 | 8 | 1.0000 | 8 | 13 | 0.2789 | 15 | 13 | 0.6312 |
| Chemotherapy assessment |  |  |  |  |  |  |  |  |  |
| resistance | 12 | 24 |  | 13 | 22 |  | 11 | 22 |  |
| sense | 18 | 6 | 0.0033* | 16 | 8 | 0.0352* | 24 | 14 | 0.0174* |
| Histological grade |  |  |  |  |  |  |  |  |  |
| I | 3 | 1 |  | 1 | 0 |  | 4 | 3 |  |
| II | 16 | 12 |  | 20 | 24 |  | 17 | 16 |  |
| III | 11 | 17 | 0.2397 | 8 | 6 | 0.4420 | 14 | 17 | 0.7978 |
| ER |  |  |  |  |  |  |  |  |  |
| negative | 6 | 11 |  | 11 | 7 |  | 18 | 14 |  |
| positive | 24 | 19 | 0.2516 | 18 | 23 | 0.2668 | 17 | 22 | 0.3442 |
| PR |  |  |  |  |  |  |  |  |  |
| negative | 12 | 13 |  | 14 | 10 |  | 25 | 22 |  |
| positive | 18 | 17 | 1.0000 | 15 | 20 | 0.2949 | 10 | 14 | 0.4537 |
| HER2 |  |  |  |  |  |  |  |  |  |
| negative | 14 | 17 |  | 9 | 8 |  | 7 | 9 |  |
| positive | 16 | 13 | 0.6058 | 20 | 22 | 0.7787 | 28 | 27 | 0.7775 |
| P53 |  |  |  |  |  |  |  |  |  |
| negative | 14 | 10 |  | 9 | 11 |  | 9 | 10 |  |
| positive | 16 | 20 | 0.4296 | 20 | 19 | 0.7847 | 26 | 26 | 1.0000 |
| Ki-67 |  |  |  |  |  |  |  |  |  |
| ≤14% | 11 | 5 |  | 8 | 5 |  | 13 | 9 |  |
| >14% | 19 | 25 | 0.1432 | 21 | 25 | 0.3604 | 22 | 27 | 0.3121 |
| Molecular subtype |  |  |  |  |  |  |  |  |  |
| HER2-overexpress | 6 | 4 |  | 6 | 9 |  | 13 | 11 |  |
| Basal-like | 2 | 5 |  | 1 | 2 |  | 2 | 5 |  |
| Luminal A | 15 | 9 |  | 9 | 5 |  | 5 | 5 |  |
| Luminal B | 7 | 12 | 0.2122 | 13 | 14 | 0.5525 | 15 | 15 | 0.6965 |

*Significance different AC: Anthracycline and Cyclophosphamide; CTF: Cyclophosphamide, Taxol and Fluorouracil; AT: Anthracycline and Taxol; CMF: Cyclophosphamide, Methotrexate and Fluorouracil.

**Table S4:**

**Association of clinical characteristics of patients with miR-214-3p** expression

|  | Training set (N=60) | | | Internal testing set (N=59) | | | Independent set (N=71) | | |
| --- | --- | --- | --- | --- | --- | --- | --- | --- | --- |
|  | Low-level (n=30) | High-level  (n=30) | *P* value | Low-level  (n=29) | High-level  (n=30) | *P* value | Low-level  (n=35) | High-level  (n=36) | *P* value |
| Age |  |  |  |  |  |  |  |  |  |
| ≤40  41-60 | 4  22 | 5  17 |  | 10  14 | 6  22 |  | 6  23 | 10  20 |  |
| ≥61 | 4 | 8 | 0.3525 | 5 | 2 | 0.1321 | 6 | 6 | 0.5501 |
| Lymph node  metastasis |  |  |  |  |  |  |  |  |  |
| absent | 5 | 9 |  | 11 | 3 |  | 10 | 14 |  |
| present | 25 | 21 | 0.3604 | 18 | 27 | 0.0153* | 25 | 22 | 0.4537 |
| T stage |  |  |  |  |  |  |  |  |  |
| T1 | 5 | 6 |  | 8 | 9 |  | 3 | 7 |  |
| T2 | 21 | 21 |  | 15 | 16 |  | 23 | 22 |  |
| T3 | 4 | 3 | 0.8897 | 6 | 5 | 0.9208 | 9 | 7 | 0.3948 |
| N stage |  |  |  |  |  |  |  |  |  |
| N0 | 5 | 9 |  | 11 | 3 |  | 10 | 14 |  |
| N1 | 11 | 5 |  | 4 | 4 |  | 10 | 7 |  |
| N2 | 10 | 7 |  | 7 | 14 |  | 10 | 6 |  |
| N3 | 4 | 9 | 0.1194 | 7 | 9 | 0.0676 | 5 | 9 | 0.3441 |
| TNM stage |  |  |  |  |  |  |  |  |  |
| I | 1 | 2 |  | 3 | 0 |  | 4 | 2 |  |
| II | 12 | 9 |  | 10 | 7 |  | 13 | 16 |  |
| III | 16 | 16 |  | 15 | 23 |  | 18 | 18 |  |
| IV | 1 | 3 | 0.6233 | 1 | 0 | 0.1023 |  |  | 0.6178 |
| Chemotherapy drug |  |  |  |  |  |  |  |  |  |
| AC | 7 | 4 |  | 1 | 3 |  | 2 | 1 |  |
| CTF | 5 | 13 |  | 6 | 7 |  | 1 | 1 |  |
| AT | 17 | 12 |  | 21 | 18 |  | 30 | 33 |  |
| CMF | 1 | 1 | 0.1553 | 1 | 2 | 0.6538 | 2 | 1 | 0.8505 |
| Chemotherapy  cycles |  |  |  |  |  |  |  |  |  |
| ≤4 | 20 | 25 |  | 16 | 22 |  | 25 | 18 |  |
| >4 | 10 | 5 | 0.2326 | 13 | 8 | 0.1799 | 10 | 18 | 0.0898 |
| Chemotherapy assessment |  |  |  |  |  |  |  |  |  |
| resistance | 13 | 23 |  | 11 | 24 |  | 10 | 23 |  |
| sense | 17 | 7 | 0.0169* | 18 | 7 | 0.0014* | 25 | 13 | 0.0042* |
| Histological grade |  |  |  |  |  |  |  |  |  |
| I | 3 | 1 |  | 0 | 1 |  | 1 | 6 |  |
| II | 10 | 18 |  | 20 | 24 |  | 13 | 20 |  |
| III | 17 | 11 | 0.1077 | 9 | 5 | 0.2879 | 21 | 10 | 0.1484 |
| ER |  |  |  |  |  |  |  |  |  |
| negative | 11 | 6 |  | 10 | 8 |  | 19 | 13 |  |
| positive | 19 | 24 | 0.2516 | 19 | 22 | 0.5796 | 16 | 23 | 0.1554 |
| PR |  |  |  |  |  |  |  |  |  |
| negative | 15 | 10 |  | 12 | 12 |  | 25 | 22 |  |
| positive | 15 | 20 | 0.2949 | 17 | 18 | 1.0000 | 10 | 14 | 0.4537 |
| HER2 |  |  |  |  |  |  |  |  |  |
| negative | 14 | 17 |  | 6 | 11 |  | 9 | 7 |  |
| positive | 16 | 13 | 0.6058 | 23 | 19 | 0.2516 | 26 | 29 | 0.5798 |
| P53 |  |  |  |  |  |  |  |  |  |
| negative | 14 | 10 |  | 9 | 11 |  | 8 | 11 |  |
| positive | 16 | 20 | 0.4296 | 20 | 19 | 0.7847 | 27 | 25 | 0.5936 |
| Ki-67 |  |  |  |  |  |  |  |  |  |
| ≤14% | 8 | 8 |  | 5 | 8 |  | 13 | 9 |  |
| >14% | 22 | 22 | 1.0000 | 24 | 22 | 0.5321 | 22 | 27 | 0.3121 |
| Molecular subtype |  |  |  |  |  |  |  |  |  |
| HER2-overexpress | 8 | 2 |  | 6 | 9 |  | 13 | 11 |  |
| Basal-like | 3 | 4 |  | 1 | 2 |  | 6 | 1 |  |
| Luminal A | 11 | 13 |  | 9 | 5 |  | 5 | 5 |  |
| Luminal B | 8 | 11 | 0.2229 | 13 | 14 | 0.5525 | 11 | 19 | 0.1187 |

*Significance different AC: Anthracycline and Cyclophosphamide; CTF: Cyclophosphamide, Taxol and Fluorouracil; AT: Anthracycline and Taxol; CMF: Cyclophosphamide, Methotrexate and Fluorouracil.

**Table S5:**

**Association of clinical characteristics of patients with miR-451a expression**

|  | Training set (N=60) | | | Internal testing set (N=59) | | | Independent set (N=71) | | |
| --- | --- | --- | --- | --- | --- | --- | --- | --- | --- |
|  | Low-level (n=30) | High-level  (n=30) | *P* value | Low-level  (n=29) | High-level  (n=30) | *P* value | Low-level  (n=35) | High-level  (n=36) | *P* value |
| Age |  |  |  |  |  |  |  |  |  |
| ≤40  41-60 | 6  20 | 3  19 |  | 6  19 | 10  17 |  | 6  22 | 10  21 |  |
| ≥61 | 4 | 8 | 0.3074 | 4 | 3 | 0.5387 | 7 | 5 | 0.5110 |
| Lymph node  metastasis |  |  |  |  |  |  |  |  |  |
| absent | 6 | 8 |  | 6 | 8 |  | 11 | 13 |  |
| present | 24 | 22 | 0.7611 | 23 | 22 | 0.7611 | 24 | 23 | 0.8056 |
| T stage |  |  |  |  |  |  |  |  |  |
| T1 | 6 | 5 |  | 8 | 9 |  | 4 | 6 |  |
| T2 | 20 | 22 |  | 15 | 16 |  | 20 | 25 |  |
| T3 | 4 | 3 | 0.8483 | 6 | 5 | 0.9208 | 11 | 5 | 0.2027 |
| N stage |  |  |  |  |  |  |  |  |  |
| N0 | 6 | 8 |  | 6 | 8 |  | 11 | 13 |  |
| N1 | 8 | 8 |  | 4 | 4 |  | 10 | 7 |  |
| N2 | 7 | 10 |  | 10 | 11 |  | 5 | 11 |  |
| N3 | 9 | 4 | 0.4338 | 9 | 7 | 0.9040 | 9 | 5 | 0.2534 |
| TNM stage |  |  |  |  |  |  |  |  |  |
| I | 2 | 1 |  | 1 | 2 |  | 3 | 3 |  |
| II | 9 | 12 |  | 8 | 9 |  | 13 | 16 |  |
| III | 17 | 15 |  | 20 | 18 |  | 19 | 17 |  |
| IV | 2 | 2 | 0.8286 | 0 | 1 | 0.6867 |  |  | 0.8157 |
| Chemotherapy drug |  |  |  |  |  |  |  |  |  |
| AC | 6 | 5 |  | 4 | 0 |  | 2 | 1 |  |
| CTF | 11 | 7 |  | 6 | 7 |  | 1 | 1 |  |
| AT | 12 | 17 |  | 16 | 23 |  | 31 | 32 |  |
| CMF | 1 | 1 | 0.6059 | 3 | 0 | 0.0399* | 1 | 2 | 0.8806 |
| Chemotherapy  cycles |  |  |  |  |  |  |  |  |  |
| ≤4 | 21 | 24 |  | 20 | 18 |  | 25 | 18 |  |
| >4 | 9 | 6 | 0.5520 | 9 | 12 | 0.5889 | 10 | 18 | 0.0898 |
| Chemotherapy assessment |  |  |  |  |  |  |  |  |  |
| resistance | 23 | 13 |  | 23 | 12 |  | 23 | 10 |  |
| sense | 7 | 17 | 0.0169* | 6 | 18 | 0.0033* | 12 | 26 | 0.0019* |
| Histological grade |  |  |  |  |  |  |  |  |  |
| I | 3 | 1 |  | 1 | 0 |  | 2 | 5 |  |
| II | 13 | 15 |  | 22 | 22 |  | 16 | 17 |  |
| III | 14 | 14 | 0.5647 | 6 | 8 | 0.5302 | 17 | 14 | 0.4510 |
| ER |  |  |  |  |  |  |  |  |  |
| negative | 9 | 8 |  | 12 | 6 |  | 16 | 16 |  |
| positive | 21 | 22 | 1.0000 | 17 | 24 | 0.0946 | 19 | 20 | 1.0000 |
| PR |  |  |  |  |  |  |  |  |  |
| negative | 14 | 11 |  | 14 | 10 |  | 22 | 25 |  |
| positive | 16 | 19 | 0.6010 | 15 | 20 | 0.2949 | 13 | 11 | 0.6210 |
| HER2 |  |  |  |  |  |  |  |  |  |
| negative | 19 | 12 |  | 7 | 10 |  | 8 | 8 |  |
| positive | 11 | 18 | 0.1205 | 22 | 20 | 0.5675 | 27 | 28 | 1.0000 |
| P53 |  |  |  |  |  |  |  |  |  |
| negative | 12 | 12 |  | 11 | 9 |  | 8 | 11 |  |
| positive | 18 | 18 | 1.0000 | 18 | 21 | 0.5889 | 27 | 25 | 0.5936 |
| Ki-67 |  |  |  |  |  |  |  |  |  |
| ≤14% | 9 | 7 |  | 5 | 8 |  | 8 | 14 |  |
| >14% | 21 | 23 | 0.7710 | 24 | 22 | 0.5321 | 27 | 22 | 0.2003 |
| Molecular subtype |  |  |  |  |  |  |  |  |  |
| HER2-overexpress | 4 | 6 |  | 10 | 5 |  | 12 | 12 |  |
| Basal-like | 5 | 2 |  | 2 | 1 |  | 4 | 3 |  |
| Luminal A | 14 | 10 |  | 5 | 9 |  | 3 | 7 |  |
| Luminal B | 7 | 12 | 0.2996 | 12 | 15 | 0.3260 | 16 | 14 | 0.6014 |

*Significance different AC: Anthracycline and Cyclophosphamide; CTF: Cyclophosphamide, Taxol and Fluorouracil; AT: Anthracycline and Taxol; CMF: Cyclophosphamide, Methotrexate and Fluorouracil.
